# Comment on “*Crystallization of Supercooled Liquids: Self-Consistency Correction of the Steady-State Nucleation Rate*” by Abyzov et al., *Entropy* 2020, *22*, 558

**DOI:** 10.3390/e22090934

**Published:** 2020-08-26

**Authors:** Vitaly A. Shneidman

**Affiliations:** Department of Physics, New Jersey Institute of Technology, Newark, NJ 07102, USA; vitaly@njit.edu

**Keywords:** transient nucleation, Becker-Döring, comment

## Abstract

It is shown that in the growth region (above the critical nucleation size) the transient distributions obtained numerically from the Becker-Döring equation (BDE) by Abyzov et al., *Entropy* **2020**, *22*, 558, are in accurate correspondence with the matched asymptotic (singular perturbation) solution by Shneidman, *Sov. Phys. Tech. Phys.* **1988**, *33*, 1338. The solution is unmodified by “self-consistency” corrections which affect only the steady state rate. Sensitivity of the results to selection of a specific form of the BDE (the “nucleation model”) also is briefly discussed.

As part of a recent study by [1] transient behavior (relaxation to steady state) of the Becker-Döring nucleation equation (BDE) with parameters related to those of lithium disilicate has been examined using numerical methods. Indeed, the time-dependent BDE does not allow a closed form exact solution. Nevertheless, an asymptotically exact solution (AES) is available in the limit of a large nucleation barrier [2,3]. Since the parameters used by [1] lead to $\Delta G\left(n_{c}\right)>50\;k_{B}T$, the AES is expected to be very accurate in the domain of its applicability. Below I briefly describe the solution and present the comparison; where possible, notations which are identical to those by [1] (except for τ) will be used.

The transient flux at size *n* in the growth region (i.e., for $n-n_{c}\gg \delta n_{c}$) is given by [2,3]

(1) $$\begin{array}{c} j\left(n,t\right)=J_{\mathrm{st}}\exp\left\{-\exp\left[\frac{t_{i}\left(n\right)-t}{\tau }\right]\right\} \end{array}$$

Note two time scales τ, the “relaxation time” and $t_{i}\left(n\right)$ the “incubation time” which is larger than τ and which depends on the size *n* where the flux is observed. One has

(2) $$\begin{array}{c} \tau =\frac{\delta n_{c}^{2}}{2w^{+}\;\left(n_{c}\right)}, \end{array}$$

while $t_{i}\left(n\right)$ is expressed in terms of the “deterministic growth rate” $\dot{n}≃w^{+}\left(n\right)-w^{-}\left(n\right)$ [i.e., in terms of an integral $\int dn/\dot{n}$]. One has [3]

(3) $$\begin{array}{c} t_{i}\left(n\right)=t_{\mathrm{dec}}\left(n_{c}-\frac{\delta n_{c}}{\sqrt{2}}\right)+t_{\mathrm{gr}}\left(n_{c}+\frac{\delta n_{c}}{\sqrt{2}}\;,\;n\right) \end{array}$$

Here $t_{\mathrm{dec}}$ is the positive decay time of a subcritical cluster with indicated initial size; $t_{\mathrm{gr}}$ is the growth time for a supercritical cluster with initial size $n_{c}+\frac{\delta n_{c}}{\sqrt{2}}$ to reach the size *n*. The experimentally obseved “induction time” (also, “time lag”) which is defined as $t_{\mathrm{ind}}\left(n\right)=\int_{0}^{\infty }\left[1-j\left(n,t\right)/J_{\mathrm{st}}\right]dt$ is then given by $t_{\mathrm{ind}}\left(n\right)=t_{i}\left(n\right)+0.5772\tau$.

Above is the general solution. To specify the model one needs an explicit growth rate $\dot{n}$. For the selection $w^{+}\propto n^{2/3}$ by [1] one has

(4) $$\begin{array}{c} \dot{n}=\frac{dn}{dR}\dot{R}\;,\;\dot{R}=\frac{R_{c}}{\tau }\frac{k_{B}T}{\Delta \mu }\left\{1-\exp\left[-\frac{\Delta \mu }{k_{B}T}\left(1-\frac{R_{c}}{R}\right)\right]\right\} \end{array}$$

(which is the “Hertz-Knudsen” growth rate in vapor condensation context); *R* and *n* are related by $\left(4/3\right)\pi R^{3}=nd_{0}^{3}$. The decay and growth contributions to the incubation time $t_{i}\left(n\right)$ in Equation (3) now can be directly evaluated:

(5) $$\begin{array}{c} t_{i}\left(n\right)=\int_{1}^{n_{c}-\frac{\delta n_{c}}{\sqrt{2}}}\frac{dn}{-\dot{n}}+\int_{n_{c}+\frac{\delta n_{c}}{\sqrt{2}}}^{n}\frac{dn^{\prime }}{{\dot{n}}^{\prime }} \end{array}$$

The “self consistency” corrections discussed by [1] do not change the rate $\dot{n}$ and thus do not affect the transient part of the AES.

In the growth region the distribution function f(n,t) is given by $j\left(n,t\right)/\dot{n}$. For comparison with numerics by [1] it is convenient to express the distribution as a function of radius, i.e., f(R,t)=f(n,t)dn/dR which tends to a constant at large *R*. Results of comparison are shown in Figure 1. As mentioned, the numerical accuracy of the AES is due to large values of the barrier $\Delta G\left(n_{c}\right)$ compared to $k_{B}T$.

In addition to the specific BDE considered by [1], other versions of the general BDE (other “models”) are discussed in literature in connection with transient nucleation in lithium disilicate. Such models differ by selection of the attachment rate $w^{+}\left(n\right)$, or by the mathematical form, continuous vs. discrete, of the nucleation master equation. The double exponential transient shape in Equation (1) remains unchanged, which allows a robust determination of τ from experimental data [3]. Otherwise, selection of another model leads to a different $\dot{n}$ compared to Equation (4), affecting the incubation time $t_{i}\left(n\right)$. For example, the “Turnbull-Fischer” model leads to $\dot{R}=2R_{c}k_{B}T/\left(\tau \Delta \mu \right)\sinh\left[\Delta \mu /\left(2k_{B}T\right)\left(1-R_{c}/R\right)\right]$ [4,5,6]. In the limit of small $\Delta \mu /k_{B}T$ this growth rate, as well as the one given by Equation (4) tend to a simpler $\dot{R}=R_{c}/\tau \left(1-R_{c}/R\right)$ which is consistent with continuous “Zeldovich-Frenkel” (ZF) version of the BDE. In that case the growth and decay integrals can be evaluated in terms of elementary functions, and one has [3]:

(6) $$\begin{array}{c} t_{i}^{ZF}\left(n\right)=\tau \left[\ln\frac{6\;\Delta G\left(n_{c}\right)}{k_{B}T}-2+\frac{R}{R_{c}}+\ln\left(\frac{R}{R_{c}}-1\right)\right] \end{array}$$

with explicit separation of the barrier- and the size-dependences. In the context of “self-consistent” correction by [1], note that the full barrier $\Delta G\left(n_{c}\right)$, rather than its reduced value $\Delta G\left(n_{c}\right)-\Delta G\left(1\right)$ enters the above expression (and thus the correction affects only $J_{st}$). The logarithmic dependence on the barrier in Equation (6) is expected to be adequate for parameters used by Abyzov et al. For the size dependence however, due to large values of $\Delta \mu /k_{B}T$ one needs the general Equations (3) and (4) in order to achieve the level of accuracy demonstrated in Figure 1.

## Figures and Tables

**Figure 1:**
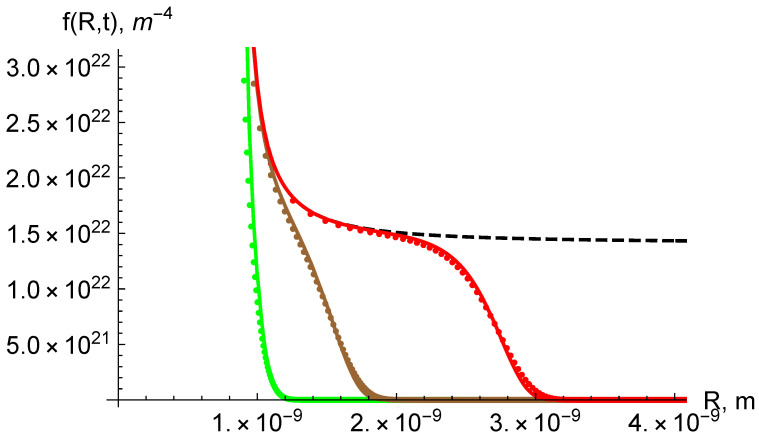
Comparison of the asymptotic solution [2,3] (lines) with numerics by [1] (symbols) at different times: left *t* = 2284 s, middle *t* = 5710 s, right *t* = 11,420 s. Dashed line is the steady state distribution in the growth region, $J_{\mathrm{st}}/\dot{R}$. No matching parameters were used.
